# Editorial: Gene, Cell and Protein Replacement Therapy for Genetic Muscle, Bone and Skin Disorders

**DOI:** 10.3389/fgene.2021.771611

**Published:** 2021-10-18

**Authors:** Holm Schneider, Michele De Luca

**Affiliations:** ^1^ Department of Pediatrics, University Hospital Erlangen, Friedrich-Alexander University Erlangen-Nürnberg, Erlangen, Germany; ^2^ Centre for Regenerative Medicine “Stefano Ferrari”, University of Modena and Reggio Emilia, Modena, Italy

**Keywords:** gene therapy, myogenic cell transplantation, protein replacement, ectodermal dysplasia, duchenne muscular dystrophy (DMD), epidermolysis bullosa

Over the past decade, various cell and gene therapy approaches have led to advanced therapy medicinal products that offer tremendous hope for patients suffering from rare genetic diseases. This especially applies to conditions affecting the musculoskeletal system or the skin, which represent the largest organs in the human body. The impairment of muscles, bone or skin due to a genetic abnormality usually has a large impact on the whole organism, and some severe hereditary conditions, such as Duchenne muscular dystrophy, are not particularly rare.

In this research topic on novel therapies, we aimed to highlight promising recent developments, challenges, and interdisciplinary scientific questions. We encouraged our colleagues to submit relevant articles of all types and included very different contributions, ranging from original research findings in a mouse model of human disease and with patient-derived induced pluripotent stem cells (iPSC) to a phase II/III clinical trial protocol.

The introductory perspective article by Koch and Koster outlines the rationale of studying rare diseases and the recent approaches they and others are applying to tackle the complexity of two developmental disorders that belong to the group of ectodermal dysplasias. They discuss the challenges for researchers in this field and focus on correcting phenotypes in the most severely affected tissues, such as wounded skin, by cell therapy strategies using genetically corrected iPSC.

Boyer et al. review myogenic cell transplantation for various genetic and acquired muscle disorders including Duchenne muscular dystrophy, oculo-pharyngeal muscular dystrophy, mitochondrial myopathies, inclusion body myositis, and sphincter incontinence. This paper provides deep insights into common or disease-specific problems that have so far limited the clinical success of myogenic cell therapy. It highlights the need for scientific, pharmaceutical and regulatory communities “to join forces and work together to produce mutual guidelines for preclinical core requirements” as well as for openness to innovative study designs in order to facilitate the translation of promising experimental results into clinical trials. This is also true for an unprecedented strategy to treat the most frequent ectodermal dysplasia (Schneider et al., 2018; Körber et al., 2020) which is based on the prenatal replacement of a missing protein, the signaling molecule ectodysplasin A1 (EDA1). For ectodermal dysplasias and some other developmental disorders, temporary protein replacement actually represents a more promising approach than cell or gene therapy attempts. An original research article by Kossel et al. illustrates this point with data showing that disturbed vertebral bone development in EDA1-deficient mice can be corrected by timely EDA1 replacement. It emphasizes tissue-specific requirements for the timing of interventions and an opportune way to overcome drug-targeting hurdles.

The two final chapters of this research topic address new gene and cell therapy approaches to treat epidermolysis bullosa, a severe genodermatosis characterized by blistering of the skin upon minor mechanical trauma. Koller and Bauer discuss emerging treatment concepts based on gene replacement or genome editing, while De Rosa et al. present the protocol of a planned clinical trial of combined cell and gene therapy, which was prompted by the successful treatment of two adults and one severely affected child with junctional epidermolysis bullosa (Hirsch et al., 2017).
